# Clinical Holistic Medicine: Holistic Rehabilitation

**DOI:** 10.1100/tsw.2005.37

**Published:** 2005-04-05

**Authors:** Søren Ventegodt, Mark Gringols, Joav Merrick

**Affiliations:** ^1^ Nordic School of Holistic Medicine and Quality of Life Research Center, Teglgårdstræde 4-8, DK-1452 Copenhagen K, Denmark; ^2^ Clalit Health Services and National Institute of Child Health and Human Development, Ben Gurion University, Beer-Sheva, Israel; ^3^ National Institute of Child Health and Human Development, Faculty of Health Sciences, Ben Gurion University of the Negev, Beer-Sheva and Office of the Medical Director, Division for Mental Retardation, Ministry of Social Affairs, Jerusalem, Israel

**Keywords:** quality of life, QOL, philosophy, human development, holistic medicine, public health, holistic health, holistic process theory, life mission theory, group therapy, Denmark

## Abstract

Quality of life, health, and ability are often lost at the same time and most often in one decaying existential movement over 5 or 10 years. This “lost life” is mostly too slow to be felt as life threatening, but once awakened to reality, it provokes the deepest of fears in patients: the fear of death itself and destruction of our mere existence. The horrible experience of having “lost life”, often without even noticing how it happened, can be turned into a strong motivation for improvement. Personal development is about finding the life deeply hidden within in order to induce revitalization and rehabilitation. Rehabilitation is about philosophy of life with the integration of the repressed painful feelings and emotions from the past and the letting go of the associated negative beliefs and decisions. The holistic medical toolbox builds on existential theories (the quality of life theories, the life mission theory, the theory of character, the theory of talent, and the holistic process theory) and seems to have the power to rehabilitate the purpose of life, the character of the person, and fundamental existential dimensions of man: (1) love; (2) strength of mind, feelings, and body; and 3) joy, gender, and sexuality; allowing the person once again to express and realize his talents and full potential. The principles of rehabilitation are not very different from other healing, but the task is often more demanding for the holistic physician as the motivation and resources often are very low and the treatment can take many years.

## INTRODUCTION

Holistic rehabilitation[1,2,3] is revitalization, which actually means resuscitation, and that is exactly what it is about. Rehabilitation is for patients who need a new and radically improved life. A patient in need of rehabilitation may feel that he or she has reached the end of his or her old life or, in other words, the patient is emotionally or existentially dead. We believe that a patient with such an experience of life is in serious difficulty and at risk of dying in the near future (for instance, from excessive drinking or suicide) if the rehabilitation project fails. On the other hand, the patient is often motivated to take a giant step forward with appropriate guidance.

The existential healing project, in all its difficult simplicity, is about teaching the patient the following: Life is your own responsibility, so you have to take fate into your own hands to return from the abyss that you are facing. You must find out what you want to do with your life, your purpose of life[4], and your personal character[5]. Although their lives seem to have gone completely off track and the future seems hopeless, it is our job as physicians and therapists to convince our patients that this is not the case and show them the road back to life. The road is bumpy, but often also fantastic and wonderful. Our firm conviction that life is not over, but merely lost, often inspires enough hope in patients to make them embark on their journey back to life.

## CLINICAL HOLISTIC MEDICINE

The life mission theory[4,5,6,7,8,9,10] states that everybody has a purpose of life or huge talent. Happiness comes from living this purpose and succeeding in expressing the core talent in your life. To do this, it is important to develop as a person into what is known as the natural condition, a condition where the person knows himself and uses all his efforts to achieve what is most important for him. The holistic process theory of healing[11,12,13,14] and the related quality of life theories[15,16,17] state that the return to the natural state of being is possible whenever the person gets the resources needed for existential healing. The resources needed are “holding” in the dimensions of awareness, respect, care, acknowledgment, and acceptance with support and processing in the dimensions of feeling, understanding, and letting go of negative attitudes and beliefs. The preconditions for the holistic healing to take place are trust and the intention for healing to take place. Existential healing is not a local healing of any tissue, but a healing of the wholeness of the person, making him much more resourceful, loving, and knowledgeable of himself, his own needs, and his wishes. In letting go of negative attitudes and beliefs, the person returns to a more responsible existential position and an improved quality of life. The philosophical change of the person healing is often a change towards preferring difficult problems and challenges instead of avoiding difficulties in life[18,19,20,21,22,23,24,25]. The person who becomes happier and more resourceful often also becomes more healthy, more talented, and more able to function[26,27,28].

## CREATING A NEW AND BETTER LIFE

Many people feel as though they are drifting aimlessly. It is as if they are not in control of their direction in life, but merely have to submit to the caprices of fortune. “I have met my fate,” the patient says when everything has fallen apart. There is some truth in that because was this person not heading towards this very wasteland that he or she is currently in?

The concept of fate has been misused, but it is a sound concept basically. Fate is our direction through life and many people sense a sinister undertone in life indicating that they are moving towards an unfortunate place. This forewarning has not been taken seriously by the rational mind. Many people in the western world believe in chance, but scientific studies of the correlation between lifestyle and illness indicate that we are indeed on an existential course that, in time, will lead us to a good or perhaps a bad place. Fate seems to be a fact of life. We shape our own personal future by the way we relate to life in the present — in a constructive or destructive manner. Let us look at a simple example and a more complex one.

> **Male, aged 47 years, and overweight:** BP check: 145/100, OK. Weight today: 138 kg. A brief discussion about weight loss strategies – taking hold – letting go – both roads can lead to the intended destination.

Obesity is related to eating patterns that are, in turn, related to self-esteem and self-appraisal, more precisely to the quality of self-care and contact with body and soul. The existential problems, of which obesity is a symptom, are therefore fundamental and can only be resolved with a whole-hearted effort. There are two paths, however. One can solve the problem by force and control — the most obvious approach in the form of weight-loss plans — or one can let go of the fundamental disturbances that are the cause of the problem. Naturally, the latter approach is better because a causal solution is also a lasting solution. Symptomatic treatment almost invariably has a temporary effect. In the following example, the patient has met his fate, so to speak. Nevertheless, he is not beyond therapeutic reach and his life can still be put right.

> **Male, aged 65 years, overweight, diabetes, stroke:** Patient has been overweight for many years and diabetic since 1997, today's BMI: 30. Episode of TIA [transient ischaemic attack] 10 days ago. He was driving the car, his wife was in the passenger seat, he started swaying more and more and increased his speed, became angry when his wife took the wheel, was driving at more than 100 km/h, when his wife resolutely pulled the hand brake and forced the car over to the side. Then the patient became paralysed on the right side; now hardly any after-effects, except impaired sense of taste. /TIA focused around right motor cortex [cerebral cortex controlling movements of the left side of the body] or perhaps thalamus [central structure in the brain]/ Examination: BP 155/65, now well-controlled. Fasting blood glucose 6.2 in latest test, i.e. only marginally increased, we will wait and see. PLAN: Intervention according to Ornish: Has now stopped smoking. Dietary advice. Weight-loss advice – should lose some weight, ideally 1 kg a week for nine weeks. Advice on physical exercise, e.g. 1 hour of swimming daily. Conversation on values in life. EXERCISE: For next time, think about improvements in life to become healthier. N.B.: should not drive a car, for at least 3 months.

His obesity is severe and has led to Type 2 diabetes. This has not prompted the patient to lose weight despite the risk of degeneration of the eyes, vascular system, and nervous system. Once the patient suffered the minor stroke, however, he became much more motivated to do something about it, and then it was a matter of seizing the opportunity. If the patient achieves substantial weight loss, the diabetes is very likely to disappear and that would improve the patient's prospects considerably. Why do we have to be close to falling over the edge before we take it seriously?

## LIFE IS CREATED BY YOU, NOT BY COINCIDENCE

When people who believe in chance get into trouble, they are "unlucky". They do not explain their misfortune as that they have systematically lived their lives "wrongly" and thus failed themselves. The notion that we control our own fate is not particularly attractive when things go wrong, but as long as everything is fine, it is much easier to accept. On further reflection, however, this notion has a certain appeal: Once we realize that we are responsible for what happens, it becomes possible to do things differently and then to fare much better in the future. The awareness that we create our own fate through our attitudes to and actions in life — our life perspective — thus brings about the interesting possibility that we can save ourselves. It is possible for us to rescue our exhausted, lost, failed, and tormented existence and start afresh in a new and rich territory.

Life falls apart because we are thinking, deciding, and acting contrary to our own life and the life of others. We are not good to ourselves, but bad. We are not good and constructive in relation to other people, but bad and destructive. Negative attitudes and perceptions are followed by inappropriate behavior that, in turn, has unfortunate consequences. The patient has started on a downward path and will hit rock bottom unless he or she can stop it.

> **Male, aged 31 years, with dizziness caused by life crisis and stress:** Borreliosis excluded, IgG negative. Persistent dizziness for nine months, no major palpitations, patient is afraid that it will return. Excessive workload – last working week he worked three days from 8 a.m. to 11.30 p.m. – and two young children also require full attention. No time for leisure, sports, friends etc. /Dizziness of disorientation type /acute stress/. Prescribed support/relief, time off work, peace and quiet. EXERCISE: Make an outline of what takes away energy and what provides energy in life. Then new appointment.

If we dissipate our energy, we may end up lacking it. The symptoms described above are common. There are many different types of dizziness, but the disorientation type is a characteristic sign of a lack of energy. It should also be noted that the suspicion was that something was wrong with his brain; that is how poorly he felt. That does not appear to be the case. If he is able to balance his "energy accounts", the dizziness will pass.

> **Female, aged 34 years, and everything is going off the rails:** Comes in desperation – everything is going off the rails – her husband wants a divorce, they have a two-year-old child together and she herself has two children from a previous relationship, two girls aged 5 and 8 years. Is no longer able to remember things, cannot concentrate, cannot watch television, cannot find her way to places, is completely out of it. Has considered taking her own life, but no specific plans, thinks about the children. What is to happen next week when she is due to meet her husband for the first time in a long while to make arrangements for a divorce and so on? Her husband says she is always morose and negative and critical, and that is also correct. We talk about it probably being best to divorce if it is simply not working, but that it is important to find an arrangement that works for the children. She has friends to talk to. On examination: assessed as not seriously depressive, no reduction in speed of speech, no waking in the early hours, slight loss of appetite, very little lowering of mood. EXERCISE: Should compile a list of all her problems. We talk about being true to oneself and about emptying one's "internal waste bin" – but first looking at and accepting everything that is in it. Can return next week, when a plan will be made.

When the bin falls over, all the waste is plainly visible. The more unresolved and repressed problems we throw into our internal waste bin, the more full it becomes, draining our energy and resources. Our energy and emotional fullness go into the waste bin along with the repression and eventually it becomes full to the brim. The patient above became increasingly childish and unreasonable and her husband became fed up and walked out on her. The children will become the losers unless the two adults pull themselves together and turn sober in less than a split second. Now is the time to think straight. Not only do the daughters need both a mother and a father, they also need their external woman to remain friends with their internal man.

## DISCUSSION

Taking control of your fate is a major, difficult, and important decision. It is not impossible, far from it. It is difficult because it is so painful to see and acknowledge all the misery that one has caused oneself and others, while blindly and stubbornly insisting on pursuing the wrong path. Native Americans say that it is never too late to turn around if one is on the wrong path. But in the western world, many people seem to believe that the fact that one has already been following that path for so long is a good argument for continuing all the way to the bitter end.

The holistic therapist supports the patient in rediscovering himself, his constructive and affirmative soul perspective. The patient should practice good and sustainable attitudes and, in that way, establish the good relations — internally and externally — that are needed in life. When your entire life has to be rebuilt from scratch and your entire existence needs to be rehabilitated, this often requires a combination of long-term training in life philosophy, psychotherapy, and body therapy. This is because the patient's dark side — the neurosis resulting from pain in life and life-limiting decisions — is tied to the body, feelings, and mind. To recover, the patient has to find the answers to some essential questions: What is kindness and ethical behavior? How can I become useful to myself and to others? What is my personal vocation in life, my life mission? What is the meaning of life and where do enjoyment of life, vital energy, and wisdom come from? To find these answers, the patient has to become fully aware of what has happened to him and what caused him to lose his enjoyment of life, vital energy, and direction in life. Usually, our advice to the patient is along the lines of: "You need to understand the principles according to how the life works inside you. You need to understand yourself. You need to get to know yourself all over again."

> **Male, aged 37 years, depressive and dull:** First consultation: 1. His wife has decided that she wants a divorce. They are getting divorced after 12 years of marriage, and they share the 7-year-old boy so that he stays with his father for three weekends every month. The patient is satisfied with this arrangement and pleased that his wife took the initiative, as he once suggested it, which crushed her completely. 2. He is constantly tired. Probably a manifestation of depression. We go through some painful events with his own parents. He stayed with his father for one week, when he was 12 years old. Emotional pain, disappointment, hope and frustration. Angry with his father for deserting him. To return for conversation in two weeks.
>
> Second consultation: 1. Small varicose veins, which the patient has had for many years and now, as a single, wants to have removed. It is agreed that the patient can be referred for surgery when they become worse. 2. Has decided that their 7-year-old son should stay with his mother, who has found a new husband with a 10-year-old daughter. Sees him every other weekend. Feels "selfish" after having been self-effacing his entire life. We discuss that. 3. We talk about his tendency towards depression. He describes himself as "dry, bare and dull", albeit with a few bright spots. We discuss the fact that the patient exists in a better version, and he will make an effort to retrieve it for next appointment. EXERCISE: The patient has read the book "Conversations with God", which is about creating the best version of yourself; his task for the next consultation is to find the passages of the book that can help him do that.

A person who sees himself as dull can work on that. He does not have to be dull because, as a living individual, he has the alternative of being vibrant, but it takes an effort to rediscover one's life. This patient will most likely attain a whole new life, far better than the one he had previously, but it requires a big effort.

Quality of life, health, and ability are often lost at the same time, most often in one decaying existential movement over 5 or 10 years. This "loss of life" is mostly too slow to be felt as life threatening, but when it is finally realized, the awakening provokes the deepest of fears in us: the fear of death itself and the destruction of our mere existence. The horrible experience of having "lost life", often without even noticing how it happened, can be turned into a strong motivation for improvement and betterment of life. Personal development is about finding the life so deeply hidden within. It is still there, and a full revitalization and rehabilitation is often possible, much to the patient's surprise. Interestingly, success in rehabilitating a patient's quality of life, health, and ability in general normally follows the successful rehabilitation of the person's philosophy of life. This rehabilitation follows the integration of the repressed painful feelings and emotions from the past and the letting go of the associated negative beliefs and decisions.

The holistic medical toolbox[29,30,31,32,33,34,35,36,37,38,39,40,41,42,43,44,45,46,47,48,49,50,51,52,53,54] seems to have the power to rehabilitate the purpose of life[4], the character of the person[5], and fundamental existential dimensions of man: (1) love; (2) strength of mind, feelings, and body; and (3) joy, gender, and sexuality[8]; allowing the person to express and realize his talents and full potential once again.

The principles of rehabilitation are not very different from the healing of other diseases and syndromes, but the task is often more demanding for the holistic physician as the motivation and resources are often very low and the treatment can take many years.

## ACKNOWLEDGMENTS

This study was supported by grants from IMK Almene Fond. The quality of life research was approved by the Copenhagen Scientific Ethical Committee under number (KF)V.100.2123/91.

## BIOSKETCHES

**Søren Ventegodt, MD,** is the director of the Quality of Life Research Center in Copenhagen, Denmark. He is also responsible for a Research Clinic for Holistic Medicine in Copenhagen and is a popular speaker throughout Scandinavia. He has published numerous scientific or popular articles and a number of books on holistic medicine, quality of life, and quality of working life. His most important scientific contributions are the comprehensive SEQOL questionnaire, the very short QoL5 questionnaire, the integrated QOL theory, the holistic process theory, the life mission theory, and the ongoing Danish Quality of Life Research Survey, 1991–94 in cooperation with the University Hospital of Copenhagen and the late professor of pediatrics, Bengt Zachau-Christiansen, MD, PhD. E-mail: ventegodt@livskvalitet.org. Website: http://www.livskvalitet.org

**Mark Gringols, BPT, MPH,** is a physiotherapist educated at the Department of Physical Therapy, School for Allied Health Professions, Sackler School of Medicine, Tel Aviv University and Department of Epidemiology, Division of Public Health, Faculty of Health Sciences, Ben Gurion University of the Negev, Beer-Sheva, Israel. Mr. Gringols works as a clinical physiotherapist for the Clalit Health Services in Beer-Sheva and is a clinical instructor at the Recanati School for Community Health Professions, Faculty of Health Sciences, Ben Gurion University of the Negev, Beer-Sheva for students in physiotherapy during their practical clinical period in the community. E-mail: gringols@bezeqint.net

**Joav Merrick, MD, DMSc,** is professor of child health and human development affiliated with the Zusman Child Development Center, Division of Pediatrics and Community Health at the Ben Gurion University, Beer-Sheva, Israel; the medical director of the Division for Mental Retardation, Ministry of Social Affairs, Jerusalem; and founder and the director of the National Institute of Child Health and Human Development. He has numerous publications in the field of child health and human development, rehabilitation, intellectual disability, disability, health, welfare, abuse, advocacy, quality of life, and prevention. Dr. Merrick received the Peter Sabroe Child Award for outstanding work on behalf of Danish Children in 1985 and the International LEGO-Prize ("The Children's Nobel Prize") for an extraordinary contribution towards improvement in child welfare and well being in 1987. E-mail: jmerrick@internet-zahav.net. Website: www.nichd-israel.com
